# Simultaneous Statistical Inference for Epigenetic Data

**DOI:** 10.1371/journal.pone.0125587

**Published:** 2015-05-12

**Authors:** Konstantin Schildknecht, Sven Olek, Thorsten Dickhaus

**Affiliations:** 1 Weierstrass Institute for Applied Analysis and Stochastics, Berlin, Germany; 2 Ivana Türbachova Laboratory for Epigenetics, Epiontis GmbH, Berlin, Germany; 3 Institute for Statistics, University of Bremen, Bremen, Germany; Indiana University Bloomington, UNITED STATES

## Abstract

Epigenetic research leads to complex data structures. Since parametric model assumptions for the distribution of epigenetic data are hard to verify we introduce in the present work a nonparametric statistical framework for two-group comparisons. Furthermore, epigenetic analyses are often performed at various genetic loci simultaneously. Hence, in order to be able to draw valid conclusions for specific loci, an appropriate multiple testing correction is necessary. Finally, with technologies available for the simultaneous assessment of many interrelated biological parameters (such as gene arrays), statistical approaches also need to deal with a possibly unknown dependency structure in the data. Our statistical approach to the nonparametric comparison of two samples with independent multivariate observables is based on recently developed multivariate multiple permutation tests. We adapt their theory in order to cope with families of hypotheses regarding relative effects. Our results indicate that the multivariate multiple permutation test keeps the pre-assigned type I error level for the global null hypothesis. In combination with the closure principle, the family-wise error rate for the simultaneous test of the corresponding locus/parameter-specific null hypotheses can be controlled. In applications we demonstrate that group differences in epigenetic data can be detected reliably with our methodology.

## Introduction

Epigenetic mechanisms, such as deoxyribonucleic acid (DNA) methylation, constitute a central principle of gene regulation. In contrast to other forms of regulation, e. g., transcriptional or translational control, DNA methylation occurs without changing the primary DNA sequence, see [1]. It refers to the selective addition of a methyl group to the 5′-carbon of the cytosine base and occurs exclusively in the dinucleotide cytosine phosphate guanine (CpG). DNA methylation occurs non-randomly and, if the target CpGs are located in the proximity of coding regions, is often associated with inactive gene expression. Oppositely, demethylation of CpG in regulatory elements is commonly accompanied by activation of expression. Shifts in DNA methylation have been observed in cells for various diseases. These changes reflect the loss of tight gene regulation as often observed in cancer. Aberrant methylation is a hallmark of dysregulation of gene control, see [2].

On the other hand, substantial variations in methylation signals in tissues or body fluids may—while still disease associated—be derived from different changes. They may result from the changing abundance of specialized cells. For example, bacterial infections cause an innate immune response to infection, and consequently the number of neutrophils is abruptly escalated. This is invariably accompanied by an equivalent increase of neutrophil-specific methylation marks. Similarly, during human immunodeficiency virus infection, a drop of CD4+ T-cells is observed and along with that the CD4 T-cell specific DNA methylation signals drop when measuring patient whole blood or leukocytes. Thus, and insofar as methylation controlled genes are cell-type specific, the concept of differential DNA methylation is employed for the discrimination and quantification of cell types, as initially shown in [3] and used in [4].

In this work, we are concerned with statistical methods in order to identify differentially methylated loci, which may be disease markers, between two groups (typically: diseased versus non-diseased). Irrespective of the exact application, numerous different technologies are employed to identify specific methylation markers, see [5]. As consequence, studies remain disparate with limited comparability between different experiments. This also extends to statistical analysis, although for this aspect a more unified approach appears feasible, since for the majority of the approaches an estimate of the methylation proportion (*β*-value) at each CpG locus for each observational unit is reported. Most current studies involve the analysis of the methylation status of multiple loci separately (e. g., Illumina methylation arrays) or on a fixed sequence/pattern of loci (e. g., Methylight). Statistically, this leads to a multiple test problem. It requires a multiple statistical hypothesis test in order to find significant differences between the groups in terms of the *β*-value at each locus or the methylation status of a sequence of loci, respectively. Depending on the objective of the study different types of multiplicity correction are appropriate. In confirmatory studies one typically aims at strong control of the family-wise error rate (FWER), meaning that the probability for at least one type I error among the locus-specific tests is bounded by a pre-defined significance level *α*. In this context, a particular challenge for statistical methodology is constituted by pronounced dependencies among the *β*-values between the loci.

Such dependencies result from at least two different principles: On one hand, due to linkage disequilibrium (see [6]), physical proximity of different CpG sites may cause bivariate dependency, with an increasing distance between two loci generally resulting in lower bivariate dependency, cf. [7]. With respect to this, however, functionally relevant gene regulation may limit the linkage (both in extent and distance). In the Foxp3 gene, for example, the promotor region is demethylated in all T-cell types whereas the regulatory T-cell (Treg) specific demethylated region is fully methylated in most and fully demethylated in just one cell type; cf. [8]. On the other hand, when considering cell type specific markers, there is also a functional-biological dependency, which must be taken into account. For example, the number of overall T-cells in peripheral blood also influences (or depends on) the number of all cells and the number of, e. g., regulatory T-cells. Hence, the number of demethylated CD3-intergenic regions—present only on all T-cells—somewhat correlates with the number of demethylated glyceraldehyde-3-phosphate dehydrogenase (GAPDH) copies—present in all cells—and the number of Foxp3 demethylated gene copies—present only in Tregs.

These pronounced dependencies, at least within blocks of loci with small genomic distance, lead to conservativity of traditional multiple test procedures like the Bonferroni correction, meaning that *α* is not exhausted. For the classes of multiple test procedures considered in this work, non-exhaustion of *α* is equivalent to sub-optimal power characteristics of the multiple tests; cf. Lemma 3.1 of [9].

Furthermore, several confounding factors of DNA methylation have been identified in previous work, e. g., methylation is known to be highly correlated with age. A test for case-control methylation data addressing this dependency was developed in [10] and extended in [11].

Several parametric models for the distribution of the *β*-values have been proposed, see [12]. Their parametric nature limits their applicability in practice. A nonparametric analysis of methylation data was suggested in [13] and [14]. However, a formal notion of multiplicity adjustment is lacking in their work. In the present paper we develop a nonparametric statistical framework for tight FWER control in the context of analyzing epigenetic methylation studies, taking the described dependencies among loci into account, leading to multivariate procedures.

## Methods

Throughout the remainder, we label reported results from the literature as propositions. Our major own mathematical contribution is Theorem 1.

### Basic Model

Suppose that we have two experimental groups denoted by *A* and *B*, for instance given by a disease status. We consider *N* ∈ ℕ observational units with *n*_*A*_ observables in group *A* and *n*_*B*_ in group *B*, such that *N* = *n*_*A*_+*n*_*B*_. We assume that all *N* observables are stochastically independent and that the observations in group *i* ∈ {*A*,*B*} are realizations of independent and identically distributed (iid.) *d*-dimensional random vectors ${\text{X}}_{ik}={\left(X_{ik}^{\left(1\right)},\ldots ,X_{ik}^{\left(d\right)}\right)}^{⊤},$ where the index *i* ∈ {*A*,*B*} denotes the group and 1 ≤ *k* ≤ *n*_*i*_ indexes the *k*-th observational unit within group *i*, while the superscript denotes the coordinate. The random vectors are assumed to follow the distribution ℒ(**X**_*A*1_) = *P* or ℒ(**X**_*B*1_) = *Q*, respectively.

**Example 1 (Identifying differentially methylated CpG loci)** *Consider an epigenetic methylation dataset comprising d CpG loci*. *For each locus ℓ a methylation ratio* (*occasionally referred to as β-value*) *is defined as*

$$\begin{array}{c} X^{\left(\ell \right)}=\frac{M^{\left(\ell \right)}}{M^{\left(\ell \right)}+U^{\left(\ell \right)}}, \end{array}$$ (1)

*where* *M^(ℓ)^* (*U^(ℓ)^*) *is an intensity value for the amount of methylated (unmethylated) cells at locus ℓ, where we assume that suitable preprocessing steps have been performed prior to the statistical analysis. In previous literature the family of beta distributions has been considered as a model for the distribution of* *X^(ℓ)^*, *e. g., in [15]. However, often bimodality and skewness are encountered, questioning this parametric assumption. Notice also that numerator and denominator in Eq (1) are highly dependent. As we are not aware of a model capturing the aforementioned distributional characteristics, we propose a nonparametric approach as in [14]. An application of our general methodology to a two-sample problem involving such data is presented in Section* “*Identification of differentially methylated CpG loci*”.

**Example 2 (Group differences for immune relevant parameters)** *As a second example, consider the comparison of human colorectal tissue for two different stages of cancer as well as healthy controls. In Section* “*Association of immune cell counts with cancer*” *we analyze data from a study in which three immune relevant parameters were measured utilizing novel epigenetic markers based on methylation signatures in tissue. Since no prior information about distributional properties of these marker data are at hand, our nonparametric approach is applied, only making use of our basic model assumptions. Three two-group comparisons are made regarding differences of the immune relevant parameters between the disease stages.*

### Aim of the statistical analysis

We denote by *F*_*i*_ the cumulative distribution function (cdf) of **X**_*i*1_ with marginal cdfs $F_{i}^{\left(\ell \right)}$ for each coordinate 1 ≤ ℓ ≤ *d*. We are interested in testing two families of marginal hypotheses, say ℋ = (*H*_ℓ_:1 ≤ ℓ ≤ *d*) and ${ℋ}^{\prime }=(H_{\ell }^{\prime }:1\leq \ell \leq d)$. The family ℋ corresponds to marginal homogeneity in the sense of [16]. This means, one is interested in testing which of the coordinate-specific marginal distributions are the same in both groups *A* and *B*, i. e.,

$$\begin{array}{c} H_{\ell }:F_{A}^{\left(\ell \right)}=F_{B}^{\left(\ell \right)}\;\text{versus}\;K_{\ell }:F_{A}^{\left(\ell \right)}\neq F_{B}^{\left(\ell \right)}. \end{array}$$

The family ℋ′ corresponds to finding a particular type of coordinate-specific differences. Namely, one is interested in detecting coordinates in which there are group differences in the central tendencies of the marginal distributions. To this end, recall the definition of the relative effect in the sense of [17].

**Definition 1 (Relative effect)** *Let* *X_A_* *and* *X_B_* *denote two stochastically independent random variables which are defined on a common probability space with probability measure ℙ. Assume that* *X_A_* *and* *X_B_* *have non-degenerate distributions and denote the normalized version of their cdf, as considered in [18], by* *F_A_* *and* *F_B_*, *respectively. Then, the relative effect of* *F_A_* *with respect to* *F_B_* *is defined as*

$$\begin{array}{c} p_{AB}=\mathbb{P}\left(X_{A}<X_{B}\right)+\frac{1}{2}\mathbb{P}\left(X_{A}=X_{B}\right)=\int F_{A}dF_{B}. \end{array}$$

*For a d-variate distribution the relative effects can be defined coordinate-wise for each 1* *≤ ℓ ≤ d by*

$$\begin{array}{c} p_{AB}^{\left(\ell \right)}=\int F_{A}^{\left(\ell \right)}dF_{B}^{\left(\ell \right)}. \end{array}$$

*Let* $p_{AB}={\left(p_{AB}^{\left(1\right)},\ldots ,p_{AB}^{\left(d\right)}\right)}^{⊤}$ *denote the vector of marginal relative effects in the latter case*.

The functional *p*_*AB*_ is capturing central tendencies, i. e., whether realizations of one of the distributions are tending to larger values than the ones from the other. Hence, we let $H_{\ell }^{\prime }:p_{AB}^{\left(\ell \right)}=1/2$ with two-sided alternatives $K_{\ell }^{\prime }:p_{AB}^{\left(\ell \right)}\neq 1/2$, 1 ≤ ℓ ≤ *d*.

Let *S* ⊆ {1,…,*d*}. In the remainder, we make use of the notation

$$\begin{array}{c} H_{S}=\underset{\ell \in S}{⋂}H_{\ell },\;H_{0}=H_{\left\{1,\cdots ,d\right\}}=\overset{d}{\underset{\ell =1}{⋂}}H_{\ell }, \end{array}$$

and refer to *H*_0_ as the global hypothesis in ℋ. An analogous notation applies for intersection hypotheses in ℋ′.

### Test statistics and multiple test procedures

For the univariate nonparametric two-sample problem, i. e., for testing one particular hypothesis *H*_ℓ_, Wilcoxon’s rank sum test (or, equivalently, the Mann-Whitney *U* test) is commonly applied. We make use of multivariate generalizations described in [19] (for testing ℋ), and in [20] (for testing ℋ′).

#### Wilcoxon-Mann-Whitney (WMW) statistic

**Definition 2 (Mann-Whitney *U*-Statistic)** *For 1* *≤ ℓ ≤* *d, we let*

$$\begin{array}{c} U^{\left(\ell \right)}=\frac{1}{n_{A}n_{B}}\sum_{j=1}^{n_{A}}\sum_{k=1}^{n_{B}}\Phi^{\left(\ell \right)}\left(X_{Aj},X_{Bk}\right) \end{array}$$

*with*

$$\begin{array}{c} \Phi^{\left(\ell \right)}\left(X_{Aj},X_{Bk}\right)=𝕀\left\{X_{Aj}^{\left(\ell \right)}>X_{Bk}^{\left(\ell \right)}\right\}. \end{array}$$

Proposition 1 (cf. Theorem 2 (iii*) of [19]) *Assume that* *n*_*i*_/*N* → *τ*_*i*_ *∈ (0,1) for* *N* → ∞, *i* ∈ {*A*,*B*}. *Then, under* *H*_0_, *it holds that*

$$\begin{array}{c} U_{N}=\sqrt{N}(U^{\left(1\right)}-\frac{1}{2},\cdots ,U^{\left(d\right)}-\frac{1}{2})\overset{𝓓}{\to }{𝓝}_{d}\left(0,\Sigma \right),\;N\to \infty , \end{array}$$ (2)

*where Σ =* (*σ*_ℓ*r*_)*_1 ≤ ℓ,r ≤ d_ with entries*

$$\begin{array}{ccc} \sigma_{\ell r} & = & \frac{\zeta_{10}^{\left(\ell ,r\right)}}{\tau_{A}}+\frac{\zeta_{01}^{\left(\ell ,r\right)}}{\tau_{B}},\\ \zeta_{10}^{\left(\ell ,r\right)} & = & \zeta_{01}^{\left(\ell ,r\right)}=\text{Cov}(\Phi^{\left(\ell \right)}\left(X_{Aj},X_{Bk}\right),\Phi^{\left(r\right)}\left(X_{Aj},X_{Bk^{\prime }}\right)), \end{array}$$

*where 1* *≤ j ≤* *n*_*A*_ *and 1 ≤* *k* *≠* *k′ ≤* *n*_*B*_. *In Eq (2) and throughout*, $\overset{𝓓}{\to }$ *denotes convergence in distribution, and 𝓝*_*d*_(*μ*,*Σ*) *stands for the d-variate normal distribution with mean μ and covariance matrix Σ*.

**Corollary 1 (Theorem 9.1 in [19])** *Let* Σ^ *be a consistent estimator of Σ. Assuming that det(Σ) > 0 it holds that*

$$\begin{array}{c} W_{N}^{U}=N{\left(U_{N}-\frac{1}{2}1_{d}\right)}^{⊤}{\hat{\Sigma }}^{-1}(U_{N}-\frac{1}{2}1_{d}) \end{array}$$

*is under H_0_ asymptotically χ^2^-distributed with d degrees of freedom as N → ∞*.

#### Empirical relative effects

The empirical counterpart of the vector **p**_*AB*_ of relative effects is denoted by ${\hat{\text{p}}}_{AB}={\left({\hat{p}}_{AB}^{\left(1\right)},\ldots ,{\hat{p}}_{AB}^{\left(d\right)}\right)}^{⊤}$ with ${\hat{p}}_{AB}^{\left(\ell \right)}=\int {\hat{F}}_{A}^{\left(\ell \right)}d{\hat{F}}_{B}^{\left(\ell \right)}$, 1 ≤ ℓ ≤ *d*, where ${\hat{F}}_{i}^{\left(\ell \right)}$, given by ${\hat{F}}_{i}^{\left(\ell \right)}(x)=n_{i}^{-1}\sum_{k=1}^{n_{i}}\frac{1}{2}\left({𝕀}_{\left(-\infty ,x\right]}(X_{ik}^{\left(\ell \right)})+{𝕀}_{\left(-\infty ,x\right)}(X_{ik}^{\left(\ell \right)})\right)$, denotes the normalized version of the empirical cdf in group *i* ∈ {*A*,*B*} pertaining to coordinate ℓ. Notice that ${\hat{p}}_{AB}^{\left(\ell \right)}=1-U^{\left(\ell \right)}$ almost surely for all 1 ≤ ℓ ≤ *d*, where *U* ^(ℓ)^ is as in Definition 2, under the assumption of absolutely continuous distributions (that there is zero probability for ties).

**Proposition 2 (Theorem 3.3 in [20])** *Let V_N_ ∈ ℝ^d×d^ denote the matrix with entries*

$$\begin{array}{ccc} v_{N}^{\left(\ell ,r\right)} & = & \frac{N}{n_{A}}c_{A}^{\left(\ell ,r\right)}+\frac{N}{n_{B}}c_{B}^{\left(\ell ,r\right)},\\ c_{i}^{\left(\ell ,r\right)} & = & Cov\left(Y_{i1}^{\left(\ell \right)},Y_{i1}^{\left(r\right)}\right),i\in \left\{A,B\right\}, \end{array}$$

*with the transformed random variables* $Y_{Ak}^{\left(\ell \right)}=F_{B}^{\left(\ell \right)}(X_{Ak}^{\left(\ell \right)})$ *and* $Y_{Bk}^{\left(\ell \right)}=F_{A}^{\left(\ell \right)}(X_{Bk}^{\left(\ell \right)})$. *Assuming that V_N_ converges to a positive definite covariance matrix V as N → ∞, it holds that*

$$\begin{array}{c} T_{N}=\sqrt{N}\left({\hat{p}}_{AB}-p_{AB}\right)\overset{𝓓}{\to }{𝓝}_{d}\left(0,V\right),\;N\to \infty . \end{array}$$

Furthermore, in (4.6) of [20] a consistent estimator ${\hat{V}}_{N}$ defined via the ranks of the observations has been provided.

**Corollary 2** *Making use of Proposition 2 and a Studentization by* ${\hat{V}}_{N}$, *it follows by Slutsky’s lemma in analogy to Corollary 1 that, under* $H_{0}^{\prime }:p_{AB}=1_{d}/2$, *the statistic*

$$\begin{array}{c} W_{N}=N{\left({\hat{p}}_{AB}-\frac{1}{2}1_{d}\right)}^{⊤}{\hat{V}}_{N}^{-1}({\hat{p}}_{AB}-\frac{1}{2}1_{d}) \end{array}$$

*is asymptotically* $\chi_{d}^{2}$-*distributed as N → ∞*.

#### Closure principle

A (non-randomized) multiple testing procedure *φ* = (*φ*_1_,…,*φ*_*d*_)^⊤^ for testing ℋ or ℋ′, respectively, is a vector of measurable mappings (individual tests) from the sample space into {0,1}^*d*^. In this, the event {*φ*_ℓ_ = 1} means rejection of the ℓ-th null hypothesis *H*_ℓ_ or $H_{\ell }^{\prime }$, respectively. For given distributions *P* and *Q*, the FWER of *φ* is defined as the probability under (*P*,*Q*) of at least one type I error, i. e.,

$$\begin{array}{c} {\text{FWER}}_{\left(P,Q\right)}\left(\varphi \right)={\mathbb{P}}_{\left(P,Q\right)}(\underset{\ell \in I_{0}\left(P,Q\right)}{⋃}\left\{\varphi_{\ell }=1\right\}), \end{array}$$

where *I*_0_(*P*,*Q*) ⊆ {1,…,*d*} denotes the index set of true null hypotheses in ℋ or ℋ′, respectively. The multiple test *φ* is said to control the FWER strongly at a given level *α* ∈ (0,1), if FWER_(*P*,*Q*)_(*φ*) ≤ *α* for all possible pairs (*P*,*Q*).

One construction principle for FWER-controlling multiple tests is the closed test principle according to [21]. The general idea behind this method is to add to the system of hypotheses of interest all their intersections *H*_*S*_ or $H_{S}^{\prime }$, respectively, where *S* ∈ 2^{1,…,*d*}^. Even if these intersection hypotheses are not of scientific interest, they are tested auxiliarly in order to provide a multiplicity correction. Namely, a closed test procedure tests every such intersection hypothesis at full level *α* by an arbitrarily chosen level *α* test *φ*_*S*_ or $\varphi_{S}^{\prime }$, respectively. The adjustment for multiplicity is then performed via the decision rule that only those coordinate-specific hypotheses *H*_ℓ_ or $H_{\ell }^{\prime }$, respectively, are rejected for which all intersection hypotheses *H*_*S*_ ($H_{S}^{\prime }$) with ℓ ∈ *S* have been rejected by *φ*_*S*_ ($\varphi_{S}^{\prime }$). Thus, the price to pay for the multiplicity of the problem is that one has to perform 2^*d*^ tests. A concise description of this principle can for instance be found in Section 3.3 of [9].

**Remark 1** *Application of the closed test principle is particularly convenient in our context by noticing that the assertions of Propositions 1 and 2 and their corollaries remain valid if the respective full d-dimensional vector of test statistics is replaced by a subvector which only contains the indices in the subset S to which φ*_*S*_ *or* $\varphi_{S}^{\prime }$, *respectively, refer. In the corollaries, only the degrees of freedom of the asymptotic χ^2^-distributions have to be changed from d to ∣S∣*.

#### Resampling-based approach

The results from the previous sections can also be used to construct asymptotically pivotal statistics for usage in a resampling approach. This strategy is assumed to keep *α* more accurately for finite *N* than the asymptotic methods resulting from Corollaries 1 and 2. In [22], multivariate multiple permutation tests have been developed for more restrictive families of hypotheses than ℋ or ℋ′, namely, for families where differences of coordinate-specific functionals of *P* and *Q*, respectively, are of interest. In contrast, the relative effect depends both on *P* and on *Q*. In Theorem 1 we adapt the theory derived in [22] to the case that multivariate relative effects are of interest. Thereby, we obtain an asymptotically FWER-controlling resampling procedure based on the statistic *W*_*N*_ or $W_{N}^{U}$, respectively.

To this end, let *π* denote an arbitrary but fixed permutation of the set {1,…,*N*} and let ${\text{X}}^{\pi }=({\text{X}}_{A1}^{\pi },\ldots ,{\text{X}}_{An_{A}}^{\pi },$ ${\text{X}}_{B1}^{\pi },\ldots ,{\text{X}}_{Bn_{B}}^{\pi })$ be the matrix containing the permuted observation vectors from **X** = (**X**_*A*1_,…,**X**_*An*_*A*__, **X**_*B*1_,…,**X**_*Bn*_*B*__). We make the convention that the first *n*_*A*_ columns of **X** and **X** ^*π*^ correspond to group *A* and the remaining *n*_*B*_ columns to group *B*. Denote by *τ* = *τ*(*π*,*n*_*A*_,*n*_*B*_) the fraction of observations from group *B* within the first *n*_*A*_ columns of **X** ^*π*^, and let ${\text{p}}_{AB}^{\pi }={\text{p}}_{A^{\prime }B^{\prime }}$, where

$$\begin{array}{ccc} 𝓛(X_{A^{\prime }1}) & = & \tau Q+\left(1-\tau \right)P=P^{\prime }\;\text{and}\\ 𝓛(X_{B^{\prime }1}) & = & \left(n_{A}/n_{B}\right)\tau P+\left(1-\left(n_{A}/n_{B}\right)\tau \right)Q=Q^{\prime }. \end{array}$$

Analogously, let ${\hat{\text{p}}}_{AB}^{\pi }={\hat{\text{p}}}_{A^{\prime }B^{\prime }}({\text{X}}^{\pi })$ denote the estimator of the vector of relative effects based on the permuted data set **X** ^*π*^. A simple calculation yields that ${\text{p}}_{AB}^{\pi }=\tau (1+n_{A}/n_{B}){\text{1}}_{d}/2+[(1-\tau (1+n_{A}/n_{B})]{\text{p}}_{AB}$. Finally, let ${\text{p}}_{AB}^{\pi }=\tau (1+n_{A}/n_{B}){\text{1}}_{d}/2+[(1-\tau (1+n_{A}/n_{B})]{\hat{\text{p}}}_{AB}$.

**Theorem 1** *Under the general setup from above, assume that the sample sizes n_A_ and n_B_ fulfill the regularity assumptions given in Lemma 5.3 of [23] as N → ∞. Define the statistic*

$$\begin{array}{c} W_{N}^{\pi }=N{\left({\hat{p}}_{AB}^{\pi }-p_{AB}^{\pi }\right)}^{⊤}{\left({\hat{V}}_{N}^{\pi }\right)}^{-1}\left({\hat{p}}_{AB}^{\pi }-p_{AB}^{\pi }\right), \end{array}$$ (3)

*where* V^Nπ *denotes the estimator from (4.6) in [20] applied to **X**^π^. Then, the permutation distribution of* $W_{N}^{\pi }$ (*i. e., its discrete distribution induced by letting π be uniformly distributed on all N! possible permutations of the set {1,…,N}, while keeping the data **X** fixed*), *the cdf of which we denote by* R^NW, *satisfies*

$$\begin{array}{c} \forall t\in \left[0,\infty \right):|{\hat{R}}_{N}^{W}\left(t\right)-F\chi_{d}^{2}\left(t\right)|\overset{P}{\to }0,\;N\to \infty . \end{array}$$

A result analogous to Theorem 1 can be obtained for the statistic $W_{N}^{U}$. Based on them, an asymptotic null distribution for *W*_*N*_ or $W_{N}^{U}$, respectively, is given by its permutation distribution. This permutation distribution, in connection with Remark 1, can be used instead of $\chi_{|S|}^{2}$ in order to calibrate each test *ϕ*_*S*_ or $\phi_{S}^{\prime }$, respectively, for type I error control at level *α*.

#### Proof of Theorem 1

We approximate the conditional distribution (given **X**) of **X** ^*π*^ by an asymptotically equivalent unconditional two-groups model. To this end, denote by **Z** = (**Z**_1_,…,**Z**_*N*_) a random matrix, the columns of which are stochastically independent such that the first *n*_*A*_ columns are distributed as *P*′ and the remaining *n*_*B*_ columns are distributed as *Q*′. Following the argument of Theorem 3.5 in [20] the statistic **T**_*N*_(**Z**) has asymptotically a centered *d*-variate normal distribution with some covariance matrix which is non-degenerate for eventually all large *N* under our general assumptions. Also, we note that both ${\hat{\text{p}}}_{AB}^{\pi }$ and ${\text{p}}_{AB}^{\pi }$ consistently estimate ${\text{p}}_{AB}^{\pi }$. Applying the reasoning of Lemma 5.3 in [23], together with the continuous mapping theorem and Slutsky’s lemma, completes the proof.

**Remark 2** *In dimension d = 1, a similar Studentized permutation approach has been discussed in [24]; see also [25].*

## Results

### Computer simulations

In this section we consider the performance of the proposed tests in terms of type I error control and power. To this end we present results of computer simulations under the following model.

**Model 1** *For each coordinate* *ℓ* *∈* *{1,…,d}* *the marginal cdf* $F_{i}^{\left(\ell \right)}$, *i* *∈* {*A*,*B*}, *is the cdf of the beta distribution with shape parameters equal to* $a_{i}^{\left(\ell \right)}$ *and* $b_{i}^{\left(\ell \right)}$. *In all simulations, the value of the second shape parameter* $b_{i}^{\left(\ell \right)}$ *was fixed as* $b_{i}^{\left(\ell \right)}=4$ *for all 1 ≤ ℓ ≤ d and i ∈ {A,B}. We consider d ∈ {2,5,10} and set the values of the first shape parameter equal to* $a_{i}^{\left(\ell \right)}=3$ *in both groups for coordinates 1,…*,*d_0_*, *where* *d_0_* *denotes the number of true null hypotheses. For the remaining* *d_1_* = *d*−*d_0_* *coordinates the values of the first shape parameter in group A are taken as* $a_{A}^{\left(\ell \right)}=3$, *while in group* *B* *the corresponding values are given as* $a_{B}^{\left(\ell \right)}=3+\delta$, *where δ takes values in {0.5,1,1.5,2,2.5,3}*.

*The dependency between the marginals is modeled by the correlation matrix R of a Gaussian copula C_Σ_, where Σ is the covariance matrix originating from R and the marginal variances induced by the shape parameters. The correlation matrix R = (R_ℓ,r_) is of AR(1) structure, i. e., R_ℓ,r_ = ρ^∣ℓ−r∣^, 1 ≤ ℓ,r ≤ d, where ρ takes values in {0,0.2,0.4,0.6,0.8}. This model is motivated by interpreting coordinates as epigenetic loci and considering a decreasing strength of dependency with increasing epigenetic distance*.

First, we assessed the accuracy of the *χ* ^2^ approximation (Proposition 2 in connection with Corollary 2) and the permutation-based approximation (Theorem 1) of the null distribution of *W*_*N*_, respectively, under the global null hypothesis. The empirical type I error rate was calculated as the relative frequency of occurrences of type I errors when testing the global null hypothesis (*d*_1_ = 0), i. e.,

$$\begin{array}{c} \frac{1}{K}\sum_{k=1}^{K}𝕀\left\{\varphi^{\left(k\right)}\left(x^{\left(k\right)}\right)=1\right\}, \end{array}$$

where *φ* ^(*k*)^ denotes the test of the global hypothesis *H*_0_ in the *k*-th of *K* simulation runs and **x** ^(*k*)^ the pseudo-sample in simulation run *k*. The empirical power of the test of the global null hypothesis was calculated as the same frequency for the cases with *d*_1_ > 0.

Second, type I error control of the multiple tests employing the closure principle was assessed by the FWER. Empirical values of the FWER were calculated as the relative frequency of the occurrence of at least one type I error, i. e.,

$$\begin{array}{c} \hat{\text{FWER}}=\frac{1}{K}\sum_{k=1}^{K}𝕀\left\{\exists 1\leq j\leq d_{0}:\varphi_{j(k)}\left(x^{\left(k\right)}\right)=1\right\}, \end{array}$$

where *φ*_(*k*)_ = (*φ*_1(*k*)_,…,*φ*_*d*(*k*)_)^⊤^ stands for the multiple test in the *k*-th simulation run.

For the sample size *N*, we considered two different regimes, namely moderate (*n*_*A*_ = 20, *n*_*B*_ = 30) and large (*n*_*A*_ = 100, *n*_*B*_ = 150). Tables 1 and 2 display empirical type I error rates for testing the global hypothesis in the moderate and large sample regimes, respectively. The empirical power for testing the global hypothesis is presented in Tables 3 and 4. Finally, Table 5 displays empirical values of the FWER, both in the moderate and in the large sample regime. The nominal significance or FWER level, respectively, was set to 5% in all simulations. The permutation test was carried out as a Monte Carlo permutation test employing 9,999 randomly chosen permutations of {1,…,*N*}, together with the identity permutation.

**Table 1:** Type I error for the global hypothesis, moderate sample sizes.

	*d* = 2		*d* = 5		*d* = 10	
*ρ*	*χ* ^2^	Perm	*χ* ^2^	Perm	*χ* ^2^	Perm
0	0.0654	0.0428	0.1034	0.0432	0.2154	0.0480
0.2	0.0668	0.0432	0.1092	0.0478	0.2064	0.0408
0.4	0.0730	0.0488	0.1092	0.0482	0.2092	0.0476
0.6	0.0654	0.0426	0.1012	0.0494	0.1898	0.0468
0.8	0.0628	0.0460	0.0848	0.0410	0.1662	0.0448

Monte Carlo simulation results, based on *K* = 10,000 repetitions, regarding the type I error rate for testing the global hypothesis in the moderate sample size regime (*n*_*A*_ = 20,*n*_*B*_ = 30) for the asymptotic *χ* ^2^-based test (*χ* ^2^) and the permutation test (Perm). The data have been generated according to Model 1 with correlation parameter *ρ*. The nominal significance level was set to *α* = 5% in all simulations. The permutation test was carried out as a Monte Carlo permutation test employing 9,999 randomly chosen permutations of {1,…,*N*}, together with the identity permutation.

**Table 2:** Type I error for the global hypothesis, large sample sizes.

	*d* = 2		*d* = 5		*d* = 10	
*ρ*	*χ* ^2^	Perm	*χ* ^2^	Perm	*χ* ^2^	Perm
0	0.0527	0.0464	0.0604	0.0448	0.0734	0.0460
0.2	0.0551	0.0456	0.0554	0.0396	0.0772	0.0500
0.4	0.0543	0.0453	0.0590	0.0440	0.0792	0.0476
0.6	0.0520	0.0440	0.0526	0.0396	0.0708	0.0458
0.8	0.0547	0.0486	0.0585	0.0460	0.0640	0.0468

Monte Carlo simulation results, based on *K* = 10,000 repetitions, regarding the type I error rate for testing the global hypothesis in the large sample size regime (*n*_*A*_ = 100,*n*_*B*_ = 150) for the asymptotic *χ* ^2^-based test (*χ* ^2^) and the permutation test (Perm). The data have been generated according to Model 1 with correlation parameter *ρ*. The nominal significance level was set to *α* = 5% in all simulations. The permutation test was carried out as a Monte Carlo permutation test employing 9,999 randomly chosen permutations of {1,…,*N*}, together with the identity permutation.

**Table 3:** Power for rejecting the global hypothesis, moderate sample sizes.

	*δ*	0.5	1	1.5	2	2.5	3
*d*_1_ = 1	*χ* ^2^	0.1456	0.2696	0.4540	0.6512	0.7948	0.8984
	Perm	0.0682	0.1524	0.2948	0.4964	0.6674	0.8176
*d*_1_ = 2	*χ* ^2^	0.1702	0.3384	0.5834	0.7986	0.9152	0.9700
	Perm	0.0890	0.2016	0.4148	0.6556	0.8270	0.9314
*d*_1_ = 3	*χ* ^2^	0.1976	0.4108	0.6882	0.8780	0.9700	0.9926
	Perm	0.1008	0.2722	0.5354	0.7824	0.9178	0.9736
*d*_1_ = 4	*χ* ^2^	0.2082	0.4744	0.7768	0.9296	0.9882	0.9982
	Perm	0.1098	0.3170	0.6402	0.8592	0.9642	0.9932
*d*_1_ = 5	*χ* ^2^	0.2236	0.5182	0.8168	0.9580	0.9946	0.9992
	Perm	0.1188	0.3560	0.6894	0.9056	0.9806	0.9962

Monte Carlo simulation results, based on *K* = 10,000 repetitions, regarding the power for testing the global hypothesis in the moderate sample size regime (*n*_*A*_ = 20,*n*_*B*_ = 30) for the asymptotic *χ* ^2^-based test (*χ* ^2^) and the permutation test (Perm). The data have been generated according to Model 1 with correlation parameter *ρ* and *d* = 5. The nominal significance level was set to *α* = 5% in all simulations. The permutation test was carried out as a Monte Carlo permutation test employing 9,999 randomly chosen permutations of {1,…,*N*}, together with the identity permutation.

**Table 4:** Power for rejecting the global hypothesis, large sample sizes.

	*δ*	0.5	1	1.5	2	2.5	3
*d*_1_ = 1	*χ* ^2^	0.2574	0.7732	0.9850	0.9998	1	1
	Perm	0.2228	0.7326	0.9804	0.9996	1	1
*d*_1_ = 2	*χ* ^2^	0.3624	0.9136	0.9990	1	1	1
	Perm	0.3202	0.8938	0.9974	1	1	1
*d*_1_ = 3	*χ* ^2^	0.4494	0.9676	1	1	1	1
	Perm	0.4020	0.9524	0.9998	1	1	1
*d*_1_ = 4	*χ* ^2^	0.5250	0.9848	1	1	1	1
	Perm	0.4760	0.9804	1	1	1	1
*d*_1_ = 5	*χ* ^2^	0.5760	0.9924	1	1	1	1
	Perm	0.5258	0.9900	1	1	1	1

Monte Carlo simulation results, based on *K* = 10,000 repetitions, regarding the power for testing the global hypothesis in the large sample size regime (*n*_*A*_ = 100,*n*_*B*_ = 150) for the asymptotic *χ* ^2^-based test (*χ* ^2^) and the permutation test (Perm). The data have been generated according to Model 1 with correlation parameter *ρ* and *d* = 5. The nominal significance level was set to *α* = 5% in all simulations. The permutation test was carried out as a Monte Carlo permutation test employing 9,999 randomly chosen permutations of {1,…,*N*}, together with the identity permutation.

**Table 5:** Empirical family-wise error rates.

	*d*_1_	0	1	2	3	4
*n*_*A*_ = 20,*ρ* = 0.1	*χ* ^2^	0.050	0.056	0.060	0.061	0.065
*n*_*B*_ = 30,*δ* = 3	Perm	0.021	0.024	0.032	0.036	0.049
*n*_*A*_ = 20,*ρ* = 0.5	*χ* ^2^	0.046	0.045	0.045	0.035	0.026
*n*_*B*_ = 30,*δ* = 1	Perm	0.021	0.016	0.018	0.017	0.011
*n*_*A*_ = 100,*ρ* = 0.5	*χ* ^2^	0.028	0.030	0.033	0.029	0.024
*n*_*B*_ = 150,*δ* = 0.5	Perm	0.020	0.020	0.024	0.022	0.018

Monte Carlo simulation results, based on *K* = 5,000 repetitions, regarding the FWER for the asymptotic *χ* ^2^-based multiple test (*χ* ^2^) and the multiple permutation test (Perm). The data have been generated according to Model 1 with correlation parameter *ρ* and *d* = 5. The nominal FWER level was set to *α* = 5% in all simulations. The permutation test was carried out as a Monte Carlo permutation test employing 9,999 randomly chosen permutations of {1,…,*N*}, together with the identity permutation.

In both sample size regimes, the empirical type I error rate of the permutation test is below the desired level of 0.05, indicating its applicability even for moderate sample sizes. In contrast, the test depending on critical values from the limiting *χ* ^2^ distribution performs liberally in all simulation settings displayed in Tables 1 and 2. With increasing dimension this test even becomes more and more liberal. For example, its empirical type I error rate rises up to 20% for *d* = 10. On the other hand the stronger the dependency between the coordinates, the less liberal the *χ* ^2^-based test.

Of course, the more stringent type I error control of the permutation test, compared with the asymptotic *χ* ^2^-based test, leads to lower power, see Tables 3 and 4. However, the differences in power become smaller for increasing *δ*.

Regarding the empirical FWER (Table 5), we again observe that the permutation test keeps the level better than the *χ* ^2^-based multiple test, where level exceedances of the latter occur for large *δ* and small *ρ* > 0 in the moderate sample size regime.

### Empirical illustration

In this section, we present applications of the proposed methods to two epigenetic studies. We applied the multiple tests based on the statistics defined in Section “Test statistics and multiple test procedures” in combination with the closure principle and Remark 1.

On one hand, we re-analyzed a representative study utilizing a whole genome approach, which aimed at the discovery of novel epigenetic markers to distinguish healthy (or good prognosis) donors from those with disease (or bad prognosis). The primary statistical challenge of such studies is the high number of locus-specific tests based on a sample with a moderate number of observations. On the other hand, we re-analyzed a data set regarding three immune relevant parameters which were derived from cell type specific real-time PCR markers in previous work (see, e. g., [3]).

#### Identification of differentially methylated CpG loci

The UK Ovarian Cancer Population Study (see [26]) aimed at detecting differentially methylated loci between ovarian cancer cases and healthy controls (GEO accession number GSE19711). To this end, 274 healthy controls were compared with 131 untreated, confirmed ovarian cancer cases. Upon rigid quality control, 264 controls and 124 cases remained in the study. When applying our method, we randomly assigned 176 and 84 controls and cases, respectively, to the screening sub-sample of a two-stage selection approach (cf. [27] and references therein). We applied the univariate two-sample Wilcoxon test at each locus on the screening sample and ranked the resulting *p*-values in ascending order. The remaining 88 and 40 control and case subjects, respectively, were used for the confirmatory analysis (second step). The ten top-ranked loci from the screening stage were tested for a relative effect unequal 1/2 based on asymptotic critical values from the limit distribution (*χ* ^2^) and permutation-based critical values (Perm) on the confirmatory group. In Table 6, the results are presented as multiplicity-adjusted *p*-values. For locus 1 ≤ ℓ ≤ 10, the multiplicity-adjusted *p*-value denotes the smallest FWER level such that $H_{\ell }^{\prime }$ is rejected by the respective multiple test procedure. With both methods, all ten candidate CpG sites have a multiplicity-adjusted *p*-value below 5%, an FWER level which is often chosen in practice.

**Table 6:** Results for the first real data example.

Locus	*cg*00645579	*cg*00974864	*cg*02679745	*cg*08044694	*cg*09134726
*χ* ^2^	0.0046	0.0002	0.0002	0.0002	0.0002
Perm	0.0126	0.0029	0.0029	0.0029	0.0029
Locus	*cg*09303642	*cg*09305224	*cg*20070090	*cg*24427660	*cg*24777950
*χ* ^2^	0.0002	0.0047	0.0001	0.0002	0.0002
Perm	0.0029	0.0146	0.0029	0.0076	0.0029

Multiplicity-adjusted *p*-values of the tests for relative effects for the loci selected at the screening stage based on the asymptotic *χ* ^2^ multiple test (*χ* ^2^) and the multiple permutation test (Perm) in combination with the closure principle. The multiplicity-adjusted *p*-value for locus ℓ denotes the smallest significance level such that $H_{\ell }^{\prime }$ is rejected for the actually observed data. The permutation test was carried out as a Monte Carlo permutation test employing 9,999 randomly chosen permutations of {1,…,*N*}, together with the identity permutation.

Among the ten loci displayed in Table 6, there are two which are associated with the FUT7 gene. In turn, the FUT7 gene encodes the Alpha-(1,3)-fucosyltransferase, see [28]. This enzyme plays a role in connection with the surfaces of granulocytes, monocytes and natural killer cells.

#### Association of immune cell counts with cancer

As mentioned in Section “Basic Model” the discussed rank-based methods can be applied under almost no assumptions due to their nonparametric nature. Furthermore, our approach implicitly adapts to the dependency structure in the data via the permutation approach. Hence, it is especially well-suited for situations with highly dependent coordinates, for example resulting from the consideration of derived parameters.

Such a situation was present in [29]. In their study, a set of three pre-identified gene regions was considered. These regions have been shown to be associated with particular cell types. Namely, demethylated Foxp3 is associated with regulatory T-cells (Tregs), CD3 with all T-cells, and GAPDH with all leukocytes. From this, three immune relevant parameters were derived: the number of Tregs, the total number of T-cells (tTL) and the cellular ratio of immune tolerance (immunoCRIT). As the Tregs constitute a subclass of the tTL and the immunoCRIT is the ratio of the two other values, these three parameters are highly dependent. Nonetheless each parameter is immune relevant in its own right.

We assessed the association of the three parameters with a disease indicator for cancer, with cancerogenesis, and with cancer progression. In this context, the evaluation of the individual roles of the parameters had to be investigated. This is because cancer tolerance may be either driven by the immunoCRIT or by its individual components, i. e., the shear amount of Tregs or all T-cells. In addition, it is important to understand, even if the most important part is the immunoCRIT, which of the components drives the change during cancerogenesis. The results are presented in Table 7.

**Table 7:** Results for the second real data example.

Parameter		Treg	tTL	immunoCRIT
Cancer indicator:				
Healthy colon versus colorectal cancer	*χ* ^2^	< 10^−16^	4.926×10^−13^	< 10^−16^
	Perm	0.0001	0.0001	0.0001
Cancerogenesis:				
Healthy colon versus early stage cancer	*χ* ^2^	5.292×10^−12^	0.0024	< 10^−16^
	Perm	0.0001	0.0044	0.0001
Cancer progression:				
Early stage cancer versus late stage cancer	*χ* ^2^	0.9043	9.710×10^−5^	0.0002
	Perm	0.9044	0.0005	0.0011

Multiplicity-adjusted *p*-values of the tests for relative effects with respect to disease groups for three different immune-relevant parameters based on the asymptotic *χ* ^2^ multiple test (*χ* ^2^) and the multiple permutation test (Perm) in combination with the closure principle. The multiplicity-adjusted *p*-value for parameter ℓ denotes the smallest significance level such that $H_{\ell }^{\prime }$ is rejected for the actually observed data. The permutation test was carried out as a Monte Carlo permutation test employing 9,999 randomly chosen permutations of {1,…,*N*}, together with the identity permutation. Treg: number of regulatory T-cells, tTL: total number of T-cells, immunoCRIT: cellular ratio of immune tolerance

Our data indicate a statistically significant role of all three parameters with respect to all three endpoints, with the exception that the Treg parameter is not significantly associated with cancer progression. Thus, our multiple permutation test confirms the notion that manifestation of cancer is strongly associated with a shift in immune tolerance as monitored by Tregs, overall T-cells and the immunoCRIT. Notably, the change of the overall immunological tolerance from healthy towards cancer tissue is driven by both the number of Tregs and the overall number of T-cells. However, once a tumor is established the continuing increase of immunoCRIT-mediated tolerance along with higher tumor stages is mainly caused by a diminished overall T-cell number and not by Treg increase. Hence, while there is an undoubted dependency among these parameters, the biological mechanisms of cancer development allow for a detachment of these parameters such that individual changes of one of the parameters can be observed and statistically evaluated.

## Discussion

Epigenetic data pose their individual set of issues for their statistical interpretation, since in contrast to DNA and protein studies, they exhibit both linkage disequilibrium-type dependencies and cell type specificity issues. Hence, dependencies have to be taken into account that go beyond the linear and parametric linkage of genetic loci, and the cell-specific linkage of expression patterns.

Here, we assessed a new method to cope with these statistical issues in a general manner. We demonstrated how group differences in epigenetic data can reliably be detected. To this end, a statistical approach based on hypotheses regarding central tendencies in combination with nonparametric Studentized multivariate multiple permutation tests has been proposed. We adapted the theory of [22] such that it can be applied to the analysis of relative effects. In particular, our methodology addresses the so-called “null dilemma” in the sense of [16], because Studentization leads to asymptotically pivotal test statistics, even if the dependency structure differs between the groups. Our approach features four important characteristics for analyzing epigenetic methylation data: (i) The use of the relative effect as a functional for the definition of differential methylation allows to declare a shift in central tendencies in case of a significant finding. This is particularly important as other studies, see [2], have found that variation in DNA methylation may play an important role in the development of complex diseases like cancer. The restriction to shift alternatives, however, is convenient for the development of certain epigenetic markers; (ii) the permutation-based approach keeps the desired type I error level even for moderate sample sizes; (iii) carrying out the permutation test as a multivariate procedure implicitly adapts to the dependency structure in the data; (iv) as we mentioned in Section “Basic Model” the discussed rank-based methods can be applied under almost no assumptions on the distribution of the data.

Computer simulations revealed that the permutation-based approach keeps the type I error level more accurately than asymptotic *χ* ^2^ approximations of the distribution of Wald-type statistics, especially in cases with moderate sample sizes. The latter finding is in line with the observations from [30]. The convergence of Wald-type statistics towards their limiting *χ* ^2^ distribution is known to be slow and this problem becomes more severe for increasing dimensionality.

As indicated in the real data examples above, epigenetic studies usually involve several loci simultaneously based on a single sample. In many medical applications, the number of observations is very limited. Each of the given examples represents one extreme—but very common—experimental set-up: Microarray analyses with thousands of mildly dependent CpGs as in Example 1 bear a substantial risk of false positives, even when relatively high sample sizes are at hand. On the other end an unknown or complicated dependency structure in the data poses a statistical challenge. This issue is true for both directly adjacent CpGs, which are usually comethylated as well as when technically independent markers functionally overlap. The latter case was considered in Example 2 with the Foxp3 gene as marker for Tregs, and CD3g/d intergenic region as marker for the overall T-cells.

As usual for resampling procedures, our approach based on permutations in combination with the closure principle is computationally much more demanding than asymptotic approximations based on tabulated *χ* ^2^-quantiles. However, computations can be parallelized with respect to the subsets *S* in the closed test procedure such that the computing time can be distributed among nodes in a cluster computing system. Furthermore, efficient shortcut versions (step-down variants) of the closed test procedure can be employed; see [31] for details.

Possible extensions of our methodology comprise multi-sample problems with more than two groups, as well as the consideration of other types of limit laws (e. g., coming from extreme value theory). Finally, Edgeworth expansions as in [32] for the Wald-type statistic can prevent the costly resampling steps, at least if some concrete distributional assumptions for the observational units can be justified.

## Supporting Information

S1 Table Data for the second real data example. The table contains measurements of three immune-relevant parameters for patients in different stages of colon cancer.
(ZIP)
